# RandomForestsGLS: An R package for Random Forests for dependent data

**DOI:** 10.21105/joss.03780

**Published:** 2022-02-25

**Authors:** Arkajyoti Saha, Sumanta Basu, Abhirup Datta

**Affiliations:** 1 Departments of Statistics, University of Washington; 2 Department of Statistics and Data Science, Cornell University; 3 Department of Biostatistics, Johns Hopkins Bloomberg School of Public Health

## Abstract

With the modern advances in geographical information systems, remote sensing technologies, and low-cost sensors, we are increasingly encountering datasets where we need to account for spatial or serial dependence. Dependent observations (*y*_1_, *y*_2_, …, *y_n_*) with covariates (x_1_, ..., x_*n*_) can be modeled non-parametrically as *y_i_* = *m*(x_*i*_) + *ϵ_i_*, where *m*(x_*i*_) is mean component and *∈_i_* accounts for the dependency in data. We assume that dependence is captured through a covariance function of the correlated stochastic process *∈_i_* (second order dependence). The correlation is typically a function of “spatial distance” or “time-lag” between two observations.

Unlike linear regression, non-linear Machine Learning (ML) methods for estimating the regression function *m* can capture complex interactions among the variables. However, they often fail to account for the dependence structure, resulting in sub-optimal estimation. On the other hand, specialized software for spatial/temporal data properly models data correlation but lacks flexibility in modeling the mean function *m* by only focusing on linear models. RandomForestsGLS bridges the gap through a novel rendition of Random Forests (RF) – namely, RF-GLS – by explicitly modeling the spatial/serial data correlation in the RF fitting procedure to substantially improve the estimation of the mean function. Additionally, RandomForestsGLS leverages kriging to perform predictions at new locations for geo-spatial data.

## Statement of need

RandomForestsGLS is a statistical machine learning oriented R package for fitting RF-GLS on dependent data. The RF-GLS algorithm described in (Saha et al., 2021) involves computationally intensive linear algebra operations in nested loops which are especially slow in an interpreted language like R. RandomForestsGLS efficiently implements RF-GLS algorithm in a low-level language with a user-friendly interface in R, a popular computational software (free under GNU General Public License) in the statistics community. RandomForestsGLS focuses on fast, parallelizable implementations of RF-GLS for spatial and time series data, which includes popular choices for covariance functions for both spatial (Matérn GP) and time series (autoregressive) data. The package is primarily designed to be used by researchers associated with the fields of statistical machine learning, spatial statistics, time series analysis, and their scientific applications. A significant part of the code has already been used in Saha et al. (2021). With the combination of speed and ease-of-use that RandomForestsGLS brings to the table regarding non-linear regression analysis for dependent data, we hope to see this package being used in a plethora of future scientific and methodological explorations.

## State of the field

Several R packages implement classical RF. Most notable of them being randomForest, which implements Breiman’s Random Forests (Breiman, 2001) for Classification and Regression using the Fortran. Some of the other packages are xgboost, randomForestSRC, ranger, Rborist. For a detailed overview of it, we refer the reader to CRAN Task View: Machine Learning & Statistical Learning. To the best of our knowledge, none of these packages explicitly account for spatial and/or temporal correlation.

Classical RF has been used in geo-spatial and temporal applications (see Saha et al. (2021) for references) without making methodological adjustments to account for spatial dependencies. (CRAN Task View: Analysis of Spatial Data, CRAN Task View: Time Series Analysis). Two recent works that attempt to explicitly use spatial information in RF for prediction purposes, are Hengl et al. (2018) ( GeoMLA) and Georganos et al. (2019) ( SpatialML) (see Saha et al. (2021) for details). Both approaches try to account for the dependence structure by incorporating additional spatial covariates, which adversely affect the prediction performance in the presence of a dominant covariate effect (Saha et al. (2021)). Additionally, unlike RF-GLS, they cannot estimate covariate effects separately from the spatial effect, which can be of independent interest. The RF for temporal data, proposed in Basak et al. (2019), suffers from similar shortcomings.

## The RandomForestsGLS package

We provide a brief overview of the functionality of the package. The package vignette demonstrates with example how to use the package for non-linear regression analysis of dependent data. Specific functions are discussed in detail in the documentation of the package.

### RF to RF-GLS: Accounting for correlation structure

In classical RF, which is an average of many regression trees, each node in a regression tree is split by optimizing the CART split criterion in Breiman et al. (1984). It can be rewritten in the following way:

$$\upsilon_{n}^{CART}\left(\left(d,c\right)\right)=\frac{1}{n}\left({\left\|Y-Z^{\left(0\right)}\hat{\beta }\left(Z^{\left(0\right)}\right)\right\|}_{2}^{2}-{\left\|Y-Z\hat{\beta }\left(Z\right)\right\|}_{2}^{2}\right).$$

where **Z**^(0)^ and **Z** are the binary membership matrices for the leaf nodes of the tree before and after the potential node split. (*d,c*) denotes a potential cut (location of the split), with *d* and *c* being the cut direction (choice of the covariate) and cutoff point (value of the covariate) respectively, $\hat{\beta }\left(Z\right)$ are the leaf node representatives given by OLS estimates corresponding to a design matrix **Z** and can be written as:

$$\hat{\beta }\left(Z\right)={\left(Z^{\text{⊤}}Z\right)}^{-1}Z^{\text{⊤}}\text{y}$$

We observe that the split criterion is the difference of OLS loss functions before and after the cut with the respective design matrices of membership of the leaf nodes. We can incorporate the correlation structure of the data in the split criterion by replacing the OLS loss with GLS loss as is traditionally done in linear models. The modified split criterion can be rewritten as:

$$\upsilon_{n,Q}^{DART}\left(\left(d,c\right)\right)=\frac{1}{n}\left[{\left(Y-Z^{\left(0\right)}{\hat{\beta }}_{GLS}\left(Z^{\left(0\right)}\right)\right)}^{⊤}Q\left(Y-Z^{\left(0\right)}{\hat{\beta }}_{GLS}\left(Z^{\left(0\right)}\right)\right)-{\left(Y-Z{\hat{\beta }}_{GLS}\left(Z\right)\right)}^{⊤}Q\left(Y-Z{\hat{\beta }}_{GLS}\left(Z\right)\right)\right].$$

where **Q** is the inverse of the working covariance matrix that models the spatial/serial dependence and ${\hat{\beta }}_{GLS}\left(Z\right)$ are the leaf node representatives given by the GLS estimates corresponding to a design matrix **Z** and can be written as follows:

$${\hat{\beta }}_{GLS}\left(Z\right)={\left(Z^{\text{⊤}}QZ\right)}^{-1}Z^{\text{⊤}}Qy.$$

### Spatial Data

#### Model

We consider spatial point-referenced data with the following mixed model:

$$y_{i}=m\left({\text{x}}_{i}\right)+w\left({\text{s}}_{i}\right)+\in_{i};$$

where *y*_*i*_, x_*i*_ respectively denotes the observed response and the covariate corresponding to the *i*^*th*^ observed location s_*i*_. *m*(x_*i*_) denotes the covariate effect; spatial random effect, *w*(s) accounts for spatial dependence beyond covariates modeled using a GP, and *∈* accounts for independent and identically distributed random Gaussian noise.

#### Fitting & Prediction

Spatial random effects are modeled using a Gaussian process (GP) as is the practice. We use the computationally convenient Nearest Neighbor GP (NNGP) (Datta et al., 2016). Model parameters, if unknown, are estimated from the data (Saha et al. (2021)). Alongside predicting covariate effects for a new covariate value, we also offer spatial prediction at new locations with non-linear kriging by combining the non-linear mean estimate and spatial kriging estimate from the BRISC (Saha & Datta, 2018) package.

### Autoregressive (AR) Time Series Data

#### Model

RF-GLS can also be used for function estimation in a time series setting under autoregressive (AR) errors. We consider time series data with errors from an AR(*q*) (autoregressive process of lag *q*) process as follows:

$$y_{t}=m\left({\text{x}}_{t}\right)+e_{t};e_{t}=\sum_{i=1}^{q}\rho_{i}e_{t-i}+\eta_{t}$$

where *y*_*i*_, x_*i*_ denote the response and the covariate corresponding to the *t*^*th*^ time point, respectively, *e*_*t*_ is an AR(q) process, *η*_*t*_ denotes i.i.d. white noise and (*ρ*_1_, …, *ρ_q_*) are the model parameters that capture the dependence of *e_t_* on (*e*_*t*−1_, …, *e_t−q_*).

#### Fitting & Prediction

RF-GLS exploits the sparsity of the closed form precision matrix of the AR process for model fitting and prediction of the mean function *m*(.). If the AR model parameters (coefficients and order of autoregressive process) are unknown, the code automatically estimates them from AR models with specified lags. The prediction of covariate effect for time series data is like that of spatial data.

## Discussion

This package provides an efficient, parallel implementation of the RF-GLS method proposed in Saha et al. (2021) which accounts for correlated data with modified node splitting criteria and node representative update rule. The package accounts for spatial correlation via Matérn GP or serial autocorrelation. It provides parameter estimation for both covariance structures. Efficient implementation through C/C++ takes advantage of the NNGP approximation and scalable node splitting update rules which reduces the execution time. More details on package features, parameter estimation, and parallelization can be found in the package README. Since the computational complexity of evaluating potential splits is cubic in the number of leaf nodes for RF-GLS, but constant for standard RF, improving the computational complexity of the algorithm is of independent research interest. We also plan to implement and validate additional forms of time series dependency.

## References

[R1] Basak S, Kar S, Saha S, Khaidem L, & Dey SR (2019). Predicting the direction of stock market prices using tree-based classifiers. The North American Journal of Economics and Finance, 47, 552–567. 10.1016/j.najef.2018.06.013

[R2] Breiman L (2001). Random forests. Machine Learning, 45(1), 5–32. 10.1023/A:1010933404324

[R3] Breiman L, Friedman J, Stone CJ, & Olshen RA (1984). Classification and regression trees. CRC press. 10.1201/9781315139470

[R4] Datta A, Banerjee S, Finley AO, & Gelfand AE (2016). Hierarchical nearest-neighbor gaussian process models for large geostatistical datasets. Journal of the American Statistical Association, 111(514), 800–812. 10.1080/01621459.2015.1044091 29720777 PMC5927603

[R5] Georganos S, Grippa T, Niang Gadiaga A, Linard C, Lennert M, Vanhuysse S, Mboga N, Wolff E, & Kalogirou S (2019). Geographical random forests: A spatial extension of the random forest algorithm to address spatial heterogeneity in remote sensing and population modelling. Geocarto International, 1–16. 10.1080/10106049.2019.1595177

[R6] Hengl T, Nussbaum M, Wright MN, Heuvelink GB, & Gräler B (2018). Random forest as a generic framework for predictive modeling of spatial and spatio-temporal variables. PeerJ, 6, e5518. 10.7717/peerj.5518 30186691 PMC6119462

[R7] Saha A, Basu S, & Datta A (2021). Random forests for spatially dependent data. Journal of the American Statistical Association, just-accepted, 1–46. 10.1080/01621459.2021.1950003

[R8] Saha A, & Datta A (2018). BRISC: Fast inference for large spatial datasets using BRISC. https://CRAN.R-project.org/package=BRISC

